# The impact of food insecurity on health outcomes: empirical evidence from sub-Saharan African countries

**DOI:** 10.1186/s12889-023-15244-3

**Published:** 2023-02-15

**Authors:** Sisay Demissew Beyene

**Affiliations:** College of Business and Economics, Department of Economics, Arsi University, Asella, Ethiopia

**Keywords:** Food insecurity, Life expectancy, Infant mortality, Panel data estimations, SSA countries

## Abstract

**Background:**

Food insecurity adversely affects human health, which means food security and nutrition are crucial to improving people’s health outcomes. Both food insecurity and health outcomes are the policy and agenda of the 2030 Sustainable Development Goals (SDGs). However, there is a lack of macro-level empirical studies (Macro-level study means studies at the broadest level using variables that represent a given country or the whole population of a country or economy as a whole. For example, if the urban population (% of the total population) of XYZ country is 30%, it is used as a proxy variable to represent represent country's urbanization level. Empirical study implies studies that employ the econometrics method, which is the application of math and statistics.) concerning the relationship between food insecurity and health outcomes in sub-Saharan African (SSA) countries though the region is highly affected by food insecurity and its related health problems. Therefore, this study aims to examine the impact of food insecurity on life expectancy and infant mortality in SSA countries.

**Methods:**

The study was conducted for the whole population of 31 sampled SSA countries selected based on data availability. The study uses secondary data collected online from the databases of the United Nations Development Programme (UNDP), the Food and Agricultural Organization (FAO), and the World Bank (WB). The study uses yearly balanced data from 2001 to 2018. This study employs a multicountry panel data analysis and several estimation techniques; it employs Driscoll-Kraay standard errors (DKSE), a generalized method of momentum (GMM), fixed effects (FE), and the Granger causality test.

**Results:**

A 1% increment in people’s prevalence for undernourishment reduces their life expectancy by 0.00348 percentage points (PPs). However, life expectancy rises by 0.00317 PPs with every 1% increase in average dietary energy supply. A 1% rise in the prevalence of undernourishment increases infant mortality by 0.0119 PPs. However, a 1% increment in average dietary energy supply reduces infant mortality by 0.0139 PPs.

**Conclusions:**

Food insecurity harms the health status of SSA countries, but food security impacts in the reverse direction. This implies that to meet SDG 3.2, SSA should ensure food security.

**Supplementary Information:**

The online version contains supplementary material available at 10.1186/s12889-023-15244-3.

## Background

Food security is essential to people’s health and well-being [1]. Further, the World Health Organization (WHO) argues that health is wealth and poor health is an integral part of poverty; governments should actively seek to preserve their people’s lives and reduce the incidence of unnecessary mortality and avoidable illnesses [2]. However, lack of food is one of the factors which affect health outcomes. Concerning this, the Food Research and Action Center noted that the social determinants of health, such as poverty and food insecurity, are associated with some of the most severe and costly health problems in a nation [3].

According to the FAO, the International Fund for Agricultural Development (IFAD), and the World Food Programme (WFP), food insecurity is defined as "A situation that exists when people lack secure access to sufficient amounts of safe and nutritious food for normal growth and development and an active and healthy life" ([4]; p50). It is generally believed that food security and nutrition are crucial to improving human health and development. Studies show that millions of people live in food insecurity, which is one of the main risks to human health. Around one in four people globally (1.9 billion people) were moderately or severely food insecure in 2017 and the greatest numbers were in SSA and South Asia. Around 9.2% of the world's population was severely food insecure in 2018. Food insecurity is highest in SSA countries, where nearly one-third are defined as severely insecure [5]. Similarly, 11% (820 million) of the world's population was undernourished in 2018, and SSA countries still share a substantial amount [5]. Even though globally the number of people affected by hunger has been decreasing since 1990, in recent years (especially since 2015) the number of people living in food insecurity has increased. It will be a huge challenge to achieve the SDGs of zero hunger by 2030 [6]. FAO et al. [7] projected that one in four individuals in SSA were undernourished in 2017. Moreover, FAO et al. [8] found that, between 2014 and 2018, the prevalence of undernourishment worsened. Twenty percent of the continent's population, or 256 million people, are undernourished today, of which 239 million are in SSA. Hidden hunger is also one of the most severe types of malnutrition (micronutrient deficiencies). One in three persons suffers from inadequacies related to hidden hunger, which impacts two billion people worldwide [9]. Similarly, SSA has a high prevalence of hidden hunger [10, 11].

An important consequence of food insecurity is that around 9 million people die yearly worldwide due to hunger and hunger-related diseases. This is more than from Acquired Immunodeficiency Syndrome (AIDS), malaria, and tuberculosis combined [6]. Even though the hunger crisis affects many people of all genders and ages, children are particularly affected in Africa. There are too many malnourished children in Africa, and malnutrition is a major factor in the high infant mortality rates and causes physical and mental development delays and disorders in SSA [12]. According to UN statistics, chronic malnutrition globally accounts for 165 million stunted or underweight children. Around 75% of these kids are from SSA and South Asia. Forty percent of children in SSA are impacted. In SSA, about 3.2 million children under the age of five dies yearly, which is about half of all deaths in this age group worldwide. Malnutrition is responsible for almost one child under the age of five dying every two minutes worldwide. The child mortality rate in the SSA is among the highest in the world, about one in nine children pass away before the age of five [12].

In addition to the direct impact of food insecurity on health outcomes, it also indirectly contributes to disordered eating patterns, higher or lower blood cholesterol levels, lower serum albumin, lower hemoglobin, vitamin A levels, and poor physical and mental health [13–15]. Iodine, iron, and zinc deficiency are the most often identified micronutrient deficiencies across all age groups. A deficiency in vitamin A affects an estimated 190 million pre-schoolers and 19 million pregnant women [16]. Even though it is frequently noted that hidden hunger mostly affects pregnant women, children, and teenagers, it further affects people’s health at all stages of life [17].

With the above information, researchers and policymakers should focus on the issue of food insecurity and health status. The SDGs that were developed in 2015 intend to end hunger in 2030 as one of its primary targets. However, a growing number of people live with hunger and food insecurity, leading to millions of deaths. Hence, this study questioned what is the impact of food insecurity on people's health outcomes in SSA countries. In addition, despite the evidence implicating food insecurity and poor health status, there is a lack of macro-level empirical studies concerning the impact of food insecurity on people’s health status in SSA countries, which leads to a knowledge (literature) gap. Therefore, this study aims to examine the impact of food insecurity on life expectancy and infant mortality in SSA countries for the period ranging from 2001–2018 using panel mean regression approaches.

### Theoretical and conceptual framework

Structural factors, such as climate, socio-economic, social, and local food availability, affect people’s food security. People’s health condition is impacted by food insecurity through nutritional, mental health, and behavioral channels [18]. Under the nutritional channel, food insecurity has an impact on total caloric intake, diet quality, and nutritional status [19–21]. Hunger and undernutrition may develop when food supplies are scarce, and these conditions may potentially lead to wasting, stunting, and immunological deficiencies [22]. However, food insecurity also negatively influences health due to its effects on obesity, women's disordered eating patterns [23], and poor diet quality [24].

Under the mental health channel, Whitaker et al. [25] noted that food insecurity is related to poor mental health conditions (stress, sadness, and anxiety), which have also been linked to obesity and cardiovascular risk [26]. The effects of food insecurity on mental health can worsen the health of people who are already sick as well as lead to disease acquisition [18]. Similarly, the behavioral channel argues that there is a connection between food insecurity and health practices that impact disease management, prevention, and treatment. For example, lack of access to household food might force people to make bad decisions that may raise their risk of sickness, such as relying too heavily on cheap, calorically dense, nutrient-poor meals or participating in risky sexual conduct. In addition, food insecurity and other competing demands for survival are linked to poorer access and adherence to general medical treatment in low-income individuals once they become sick [27–30]

Food insecurity increases the likelihood of exposure to HIV and worsens the health of HIV-positive individuals [18]. Weiser et al. [31] found that food insecurity increases the likelihood of unsafe sexual activities, aggravating the spread of HIV. It can also raise the possibility of transmission through unsafe newborn feeding practices and worsening maternal health [32]. In addition, food insecurity has been linked to decreased antiretroviral adherence, declines in physical health status, worse immunologic status [33], decreased viral suppression [34, 35], increased incidence of serious illness [36], and increased mortality [37] among people living with HIV.

**Fig. 1:**
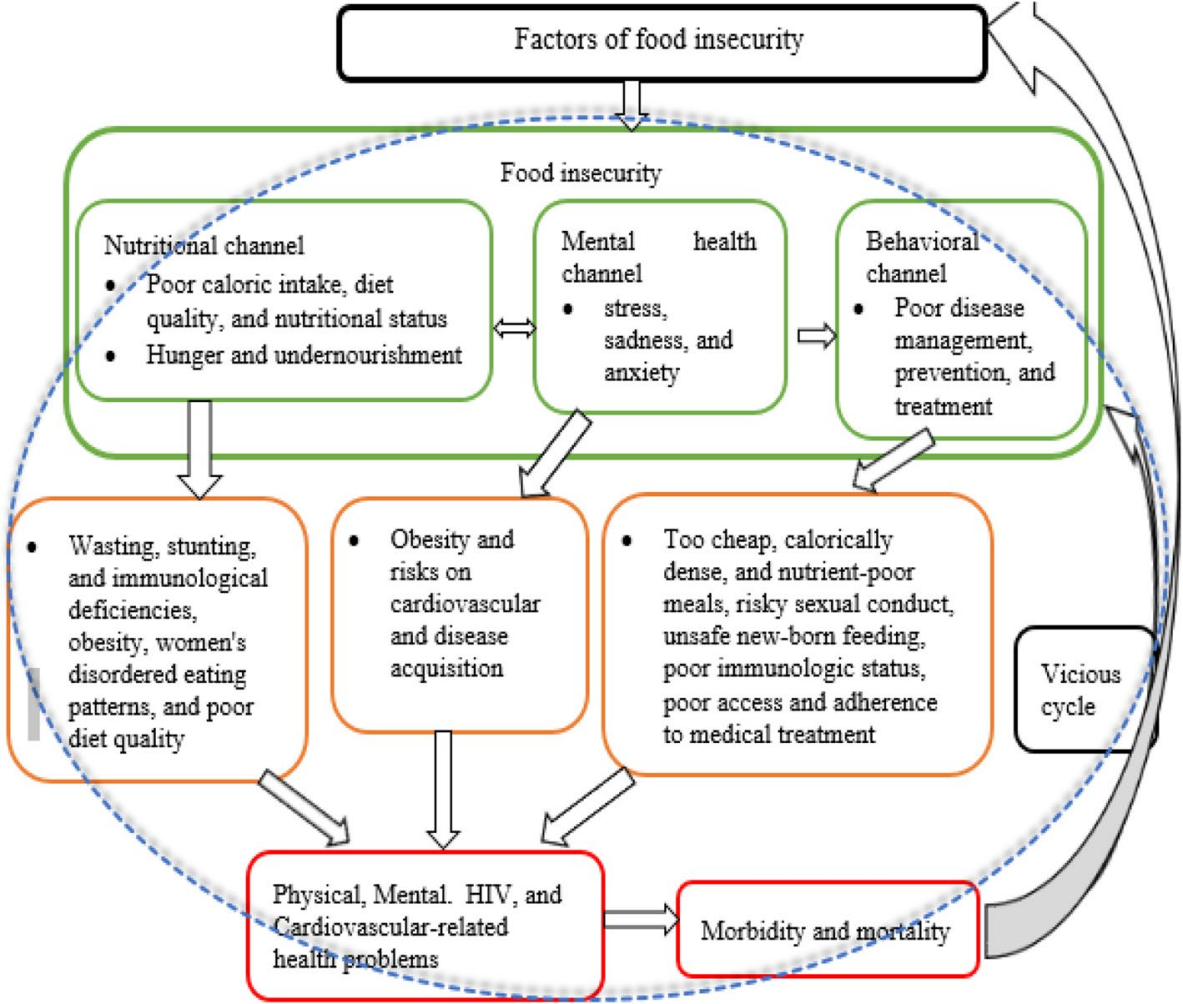
A conceptual framework of food insecurity and health. Source: Modified and constructed by the author using Weiser et al. [18] conceptual framework. Permission was granted by Taylor & Francis to use their original Figs. (2.2, 2.3, and 2.4); to develop the above figure. Permission number: 1072954

With the above theoretical relationship between target variables and since this study focuses on the impact of food insecurity on health outcomes, and not on the causes, it adopted the conceptual framework of Weiser et al. [18] and constructed Fig. 1.

Several findings associate food insecurity with poorer health, worse disease management, and a higher risk of premature mortality even though they used microdata. For instance, Stuff et al. [38] found that food insecurity is related to poor self-reported health status, obesity [39], abnormal blood lipids [40], a rise in diabetes [24, 40], increased gestational diabetes[41], increased perceived stress, depression and anxiety among women [25, 42], Human Immunodeficiency Virus (HIV) acquisition risk [43–45], childhood stunting [46], poor health [47], mental health and behavioral problem [25, 48, 49].

The above highlight micro-level empirical studies, and since the scope of this study is macro-level, Table 1 provides only the existing macro-level empirical findings related to the current study.

**Table 1:** Empirical review

Author(s)	Model applied	Scope	Results	Limitations
Uchendu [50]	T-test, multivariate, and Pearson correlation	2005–2014, 14 war-torn SSA countries	Hunger and malnutrition indicators influence life expectancy	Conventional methodology, outdated, limited sample size, and no robustness checks
Asiseh et al. [51]	FE and instrumental variable approach	For various years, middle and low-income countries	Food insecurity has a negative relationship with life expectancy	Out-dated, no basic econometric tests before estimation, no robustness checks, conventional methodology
Justice & Louis [52]	Cointegration and Vector Error Correction Mechanism	1970–2015, Nigeria	Infant mortality is negatively associated with food production	Only one country, outdated data, and conventional estimation technique
Hameed et al. [53]	GMM	2001–2018, developing Asian countries	negative correlation between food insecurity and women and child health outcome,	No basic econometric tests, no robustness checks, and conventional methodology
Banerjee et al. [54]	A Cox proportional hazards model	2005–2015, USA	Food insecurity leads to higher mortality and also cardiovascular mortality	One country and outdated data
Cassidy-Vu, et al. [55]	Multivariable linear regression	For various years, North Carolina	Food insecurity positively correlated with infant mortality	One country, unbalanced data, and conventional methodology

Source: Constructed by the author

Empirical findings in Table 1 are a few, implying a limited number of macro-level level empirical findings. Even the existing macro-level studies have several limitations. For instance, most studies either employed conventional estimation techniques or overlooked basic econometric tests; thus, their results and policy implications may mislead policy implementers. Except for Hameed et al. [53], most studies’ data are either outdated or unbalanced; hence, their results and policy implications may not be valuable in the dynamic world and may not be accurate like balanced data. Besides, some studies used limited (one) sampled countries; however, few sampled countries and observations do not get the asymptotic properties of an estimator [56]. Therefore, this study tries to fill the existing gaps by employing robust estimation techniques with initial diagnostic and post-estimation tests, basic panel econometric tests and robustness checks, updated data, a large number of samples.

## Methods

### Study setting and participants

According to Smith and Meade [57], the highest rates of both food insecurity and severe food insecurity were found in Sub-Saharan Africa in 2017 (55 and 28%, respectively), followed by Latin America and the Caribbean (32 and 12%, respectively) and South Asia (30 and 13%). Similarly, SSA countries have worst health outcomes compared to other regions. For instance, in 2020, the region had the lowest life expectancy [58] and highest infant mortality [59]. Having the above information, this study's target population are SSA countries chosen purposively. However, even though SSA comprises 49 of Africa's 55 countries that are entirely or partially south of the Sahara Desert. This study is conducted for a sample of 31 SSA countries (Angola, Benin, Botswana, Burkina Faso, Cameroon, Cabo Verde, Chad, Congo Rep., Côte d'Ivoire, Ethiopia, Gabon, The Gambia, Ghana, Kenya, Lesotho, Liberia, Madagascar, Malawi, Mali, Mauritania, Mauritius, Mozambique, Namibia, Nigeria, Rwanda, Senegal, Sierra Leone, South Africa, Sudan, Tanzania, and Togo). The sampled countries are selected based on data accessibility for each variable included in the empirical models from 2001 to 2018. Since SSA countries suffer from food insecurity and related health problems, this study believes the sampled countries are appropriate and represent the region. Moreover, since this study included a large sample size, it improves the estimator’s precision.

### Data type, sources, and scope

This study uses secondary data collected in December 2020 online from the databases of the Food and Agricultural Organization (FAO), the United Nations Development Programme (UNDP), and the World Bank (WB) (see Table 2). In addition, the study uses yearly balanced data from 2001 to 2018, which is appropriate because it captures the Millennium Development Goals, SDGs, and other economic conditions, such as the rise of SSA countries’ economies and the global financial crisis of the 2000s. Therefore, this study considers various global development programs and events. Generally, the scope of this study (sampled countries and time) is sufficient to represent SSA countries. In other words, the study has n*T = 558 observations, which fulfills the large sample size criteria recommended by Kennedy [56].

**Table 2:** Data type, sources, and measurements

Variables	Definitions and measurements	Sources
LNLEXP	Natural logarithm of life expectance measured as life expectance at birth, total (years)	WB database https://databank.worldbank.org/source/world-development-indicators#
LNINFMOR	Natural logarithm of infants dying before one-year-old measured as mortality rate, infant (per 1,000 live births)	
PRUDN	Prevalence of undernourishment measured as % of the population – prevalence of undernourishment is the percentage of the population whose habitual food consumption is insufficient to provide the dietary energy levels that are required to maintain a normally active and healthy life	
GDPPC	GDP per capita (constant 2010 US$)	
GOVEXP	Domestic general government health expenditure (% of GDP)	
URBAN	Urbanization is measured as urban population (% of the total population)	
MNSCHOOL	Mean years of schooling (years)	UNDP database http://hdr.undp.org/en/data
AVRDES	Average dietary energy supply adequacy is measured as % (3-year average), which is dietary energy supply as a percentage of the average dietary energy requirement. Each country’s or region’s average supply of calories for food consumption is normalized by the average dietary energy requirement estimated for its population to provide an index of adequacy of the food supply in terms of calories	FAO database https://knoema.com/FAOFSD2020/fao-food-security-data?location=1000180-sub-saharan-africa

Source: Constructed by the author

### The empirical model

Model specification is vital to conduct basic panel data econometric tests and estimate the relationship of target variables. Besides social factors, the study includes economic factors determining people's health status. Moreover, it uses two proxies indicators to measure both food insecurity and health status; hence, it specifies the general model as follows:

1 $$\overbrace{LNLEXP\left(LNINFMOR\right)}^{Target\;variables}=\overset{Target\;variables}{f\overbrace{(PRUND(AVRDES)},\;}\overbrace{GDPPC,\;GOVEXP,\;MNSCHOOL,\;URBAN)}^{Control\;variables}$$

The study uses four models to analyze the impact of food insecurity on health outcomes.

1A $${LNLEXP}_{it}={\alpha }_{0}+{\alpha }_{1}{PRUND}_{it}+{\alpha }_{2}{GDPPC}_{it}+{\alpha }_{3}{GOVEXP}_{it}+{\alpha }_{4}{MNSCHOOL}_{it}+{\alpha }_{5}{URBAN}_{it}+{\upeta }_{it}$$

1B $${LNLEXP}_{it}={\beta }_{0}+{\beta }_{1}{AVRDES}_{it}+{\beta }_{2}{GDPPC}_{it}+{\beta }_{3}{GOVEXP}_{it}+{\beta }_{4}{MNSCHOOL}_{it}+{\beta }_{5}{URBAN}_{it}+{v}_{it}$$

1C $${LNINFMOR}_{it}={\theta }_{0}+{\theta }_{1}{PRUND}_{it}+{\theta }_{2}{GDPPC}_{it}+{\theta }_{3}{GOVEXP}_{it}+{\theta }_{4}{MNSCHOOL}_{it}+{\theta }_{5}{URBAN}_{it}+{\varepsilon }_{it}$$

1D $${LNINFMOR}_{it}={\delta }_{0}+{\delta }_{1}{AVRDES}_{it}+{\delta }_{2}{GDPPC}_{it}+{\delta }_{3}{GOVEXP}_{it}+{\delta }_{4}{MNSCHOOL}_{it}+{\delta }_{5}{URBAN}_{it}+{\mu }_{it}$$

where LNLEXP and LNINFMOR (dependent variables) refer to the natural logarithm of life expectancy at birth and infant mortality used as proxy variables for health outcomes. Similarly, PRUND and AVRDES are the prevalence of undernourishment and average dietary energy supply adequacy – proxy and predictor variables for food insecurity.

Moreover, to regulate countries’ socio-economic conditions and to account for time-varying bias that can contribute to changes in the dependent variable, the study included control variables, such as GDPPC, GOVEXP, MNSCHOOL, and URBAN. GDPPC is GDP per capita, GOVEXP refers to domestic general government health expenditure, MNSCHOOL is mean years of schooling and URBAN refers to urbanization. Further, *n*_*it*_, *v*_*it*_, ε_*it*_, and μ_*it*_ are the stochastic error terms at period *t.* The parameters ${\alpha }_{0}, { \beta }_{0}, { \theta }_{0},{ \delta }_{0}$ refer to intercept terms and ${\alpha }_{1}-{\alpha }_{5}, {\beta }_{1}-{\beta }_{5}, { \theta }_{1}-{\theta }_{5}, and {\delta }_{1}-{\delta }_{5}$ are the long-run estimation coefficients. Since health outcomes and food insecurity have two indicators used as proxy variables, this study estimates different alternative models and robustness checks of the main results. Furthermore, the above models did not address heterogeneity problems; hence, this study considers unobserved heterogeneity by introducing cross-section and time heterogeneity in the models. This is accomplished by assuming a two-way error component for the disturbances with:

2 $$\left\{\begin{array}{c}{\upeta }_{it}\\ {\nu }_{it}\\ {\varepsilon }_{it}\\ {\mu }_{it}\end{array}=\varpi =\right.{\delta }_{i}+{\tau }_{t}+{\gamma }_{it}$$

From Eq. 2, the unobservable individual (cross-section) and unobservable time heterogeneities are described by ${\delta }_{i} and {\tau }_{t}$ (within components), respectively. Nonetheless, the remaining random error term is ${\gamma }_{it}$ (panel or between components). Therefore, the error terms in *model 1A-1D* will be substituted by the right-hand side elements of Eq. 2.

Depending on the presumptions of whether the error elements are fixed or random, the FE and RE models are the two kinds of models that will be evaluated. Equation (2) yields a two-way FE error component model, or just a FE model if the assumptions are that ${\delta }_{i} and {\tau }_{t}$ are fixed parameters to be estimated and that the random error component, ${\gamma }_{it}$, is uniformly and independently distributed with zero mean and constant variance (homoscedasticity).

Equation (2), on the other hand, provides a two-way RE error component model or a RE model if we suppose ${\delta }_{i} and {\tau }_{t}$ are random, just like the random error term, or ${\delta }_{i},{\tau }_{t}, and {\gamma }_{it}$ are all uniformly and independently distributed with zero mean and constant variance, or they are all independent of each other and independent variables [60].

Rather than considering both error components, ${\delta }_{i}, and {\tau }_{t}$, we can examine only one of them at a time (fixed or random), yielding a one-way error component model, FE or RE. The stochastic error term ${\varpi }_{it}$ in Eq. 2 will then be:

3 $${\varpi }_{it}={\delta }_{i}+{\gamma }_{it}, or$$

4 $${\varpi }_{it}={\tau }_{t}+{\gamma }_{it}$$

### Statistical analysis

This study conducted descriptive statistics, correlation analysis, and initial diagnosis tests (cross-sectional and time-specific fixed effect, outliers and influential observations, multicollinearity, normality, heteroscedasticity, and serial correlation test). Moreover, it provides basic panel econometric tests and panel data estimation techniques. For consistency, statistical software (STATA) version 15 was used for all analyses.

### Descriptive statistics and correlation analysis

Descriptive statistics is essential to know the behavior of the variables in the model. Therefore, it captures information, such as the mean, standard deviation, minimum, maximum, skewness, and kurtosis. Similarly, the study conducted Pearson correlation analysis to assess the degree of relationship between the variables.

### Initial diagnosis

#### Cross-sectional and time-specific fixed effect

One can anticipate differences arising over time or within the cross-sectional units, given that the panel data set comprises repeated observations over the same units gathered over many periods. Therefore, before estimation, this study considered unexplained heterogeneity in the models. One fundamental limitation of cross-section, panel, and time series data regression is that they do not account for country and time heterogeneity [60]. These unobserved differences across nations and over time are crucial in how the error term is represented and the model is evaluated. These unobserved heterogeneities, however, may be represented by including both country and time dummies in the regression. However, if the parameters exceed the number of observations, the estimate will fail [60]. However, in this study, the models can be estimated. If we include both country and time dummies, we may assume that the slope coefficients are constant, but the intercept varies across countries and time, yielding the two-way error components model. As a result, this study examines the null hypothesis that intercepts differ across nations and time in general.

#### Detecting outliers and influential observations

In regression analysis, outliers and influential observations may provide biased findings. Therefore, the Cooks D outlier and influential observation test was used in the study to handle outliers and influencing observations. To evaluate whether these outliers have a stronger impact on the model to be estimated, each observation in this test was reviewed and compared with Cook’s D statistic [61]. Cook distance evaluates the extent to which observation impacts the entire model or the projected values. Hence, this study tested the existence of outliers.

#### Normality, heteroscedasticity, multicollinearity, and serial correlation test

Before the final regression result, the data used for the variables were tested for normality, heteroscedasticity, multicollinearity, and serial correlation to examine the characteristics of the sample.

Regression models should be checked for nonnormal error terms because a lack of Gaussianity (normal distribution) can occasionally compromise the accuracy of estimation and testing techniques. Additionally, the validity of inference techniques, specification tests, and forecasting critically depends on the normalcy assumption [62]. Similarly, multicollinearity in error terms leads to a dataset being highly sensitive to a minor change, instability in the regression model, and skewed and unreliable results. Therefore, this study conducted the normality using Alejo et al. [62] proposed command and multicollinearity (using VIF) tests.

Most conventional panel data estimation methods rely on homoscedastic individual error variance and constant serial correlation. Since the error component is typically connected to the variance that is not constant during the observation and is serially linked across periods, these theoretical presumptions have lately reduced the applicability of various panel data models. Serial correlation and heteroskedasticity are two estimate issues frequently connected to cross-sectional and time series data, respectively. Similarly, panel data is not free from these issues because it includes cross-sections and time series, making the estimated parameters ineffective, and rendering conclusions drawn from the estimation incorrect [63]. Therefore, this study used the Wooldridge [63] test for serial correlation in linear panel models as well as the modified Wald test for heteroskedasticity.

### Basic panel econometric tests

The basic panel data econometric tests are prerequisites for estimating the panel data. The three main basic panel data tests are cross-sectional dependence, unit root, and cointegration.

#### Cross-sectional dependence (CD)

A growing body of the panel data literature concludes that panel data models are likely to exhibit substantial CD in the errors resulting from frequent shocks, unobserved components, spatial dependence, and idiosyncratic pairwise dependence. Even though the impact of CD in estimation depends on several factors, relative to the static model, the effect of CD in dynamic panel estimators is more severe [64]. Moreover, Pesaran [65] notes that recessions and economic or financial crises potentially affect all countries, even though they might start from just one or two countries. These occurrences inevitably introduce cross-sectional interdependencies across the cross-sectional unit, their regressors, and the error terms. Hence, overlooking the CD in panel data leads to biased estimates and spurious results [64, 66]. Further, the CD test determines the type of panel unit root and cointegration tests we should apply. Therefore, examining the CD is vital in panel data econometrics.

In the literature, there are several tests for CD, such as the Breusch and Pagan [67] Lagrange multiplier (LM) test, Pesaran [68] scaled LM test, Pesaran [68] CD test, and Baltagi et al. [69] bias-corrected scaled LM test (for more detail, see Tugcu and Tiwari [70]). Besides, Friedman [71] and Frees [72, 73] also have other types of CD tests (for more detail, see De Hoyos and Sarafidis [64]). This study employs Frees [72] and Pesaran [68] among the existing CD tests. This is because, unlike the Breusch and Pagan [67] test, these tests do not require infinite T and fixed N, and are rather applicable for both a large N and T. Additionally, Free’s CD test can overcome the irregular signs associated with correlation. However, it also employs Friedman [71] CD for mixed results of the above tests.

#### Unit root test

The panel unit root and cointegration tests are common steps following the CD test. Generally, there are two types of panel unit root tests: (1) the first-generation panel unit root tests, such as Im et al. [74], Maddala and Wu [75], Choi [76], Levin et al. [77], Breitung [78] and Hadri [79], and (2) the second-generation panel unit root tests, such as [66, 80–89].

The first-generation panel unit root tests have been criticized because they assume cross-sectional independence [90–93]. This hypothesis is somewhat restrictive and unrealistic, as macroeconomic time series exhibit significant cross-sectional correlation among countries in a panel [92], and co-movements of economies are often observed in the majority of macroeconomic applications of unit root tests [91]. The cross-sectional correlation of errors in panel data applications in economics is likely to be the rule rather than the exception [93]. Moreover, applying first-generation unit root tests under CD models can generate substantial size distortions [90], resulting in the null hypothesis of nonstationary being quickly rejected [66, 94]. As a result, second-generation panel unit root tests have been proposed to take CD into account. Therefore, among the existing second-generation tests, this study employs Pesaran’s [66] cross-sectionally augmented panel unit root test (CIPS) for *models 1A–1C*. The rationale for this is that, unlike other unit root tests that allow CD, such as Bai and Ng [80], Moon and Perron [87], and Phillips and Sul [84], Pesaran’s [66] test is simple and clear. Besides, Pesaran [66] is robust when time-series’ heteroscedasticity is observed in the unobserved common factor [95]. Even though theoretically, Moon and Perron [87], Choi [96] and Pesaran [66] require large N and T, Pesaran [66] is uniquely robust in small sample sizes [97]. Therefore, this study employs the CIPS test to take into account CD, and heteroskedasticity in the unobserved common factor and both large and small sample countries. However, since there is no CD in *model 1D*, this study employs the first-generation unit root tests called Levin, Lin, and Chu (LLC), Im, Pesaran, Shin (IPS) and Fisher augmented Dickey–Fuller (ADF) for *model 1D*.

#### Cointegration test

The most common panel cointegration tests when there is CD are Westerlund [98], Westerlund and Edgerton [99], Westerlund and Edgerton [100], Groen and Kleibergen [101], Westerlund’s [102] Durbin-Hausman test, Gengenbach et al. [103] and Banerjee and Carrion-i-Silvestre [104]. However, except for a few, most tests are not coded in Statistical Software (STATA) and are affected by insufficient observations. The current study primarily uses Westerlund [98] and Banerjee and Carrion-i-Silvestre [104] for *models 1A–1C*. However, to decide uncertain results, it also uses McCoskey and Kao [105] cointegration tests for *model 1C*. The rationale for using Westerlund’s [98] cointegration test is that most panel cointegration has failed to reject the null hypothesis of no cointegration due to the failure of common-factor restriction [106]. However, Westerlund [98] does not require any common factor restriction [107] and allows for a large degree of heterogeneity (e.g., individual-specific short-run dynamics, intercepts, linear trends, and slope parameters) [92, 107, 108]. Besides, its command is coded and readily available in STATA. However, it suffers from insufficient observations, especially when the number of independent variables increases. The present study employs the Banerjee and Carrion-i-Silvestre [104] and McCoskey and Kao [105] cointegration tests to overcome this limitation. The two Engle-Granger-based cointegration tests applicable when there is no CD and are widely used and available in STATA are Pedroni [109, 110] and Kao [111]. However, the Pedroni test has two benefits over Kao: it assumes cross-sectional dependency and considers heterogeneity by employing specific parameters [112]. Hence, this study uses the Pedroni cointegration test for *model 1D*.

### Panel data estimation techniques

The panel data analysis can be conducted using different estimation techniques and is mainly determined by the results of basic panel econometric tests. Thus, this study mainly employs the Driscoll-Kraay [113] standard error (DKSE) (for *models 1A* and *1B*), FE (for *model 1C*), and two-step GMM (for *model 1D*) estimation techniques to examine the impact of food insecurity on health outcomes. It also employs the Granger causality test. However, for robustness checks, it uses fully modified ordinary least squares (FMOLS), panel-corrected standard error (PCSE), and feasible generalized least squares (FGLS) methods (for *models 1A and 1B*). Moreover, it uses a random effect (RE) for *model 1C* and panel dynamic fixed effect (DFE) techniques for *model 1D*.

Even though several panel estimation techniques allow CD, most of them – such as cross-section augmented autoregressive distributed lag (CS-ARDL), cross-section augmented distributed lag (CS-DL), common correlated effects pooled (CCEP), and common correlated effects mean group (CCEMG) estimators – require a large number of observations over groups and periods. Similarly, the continuously updated fully modified (CUP-FM) and continuously updated bias-corrected (CUP-BC) estimators are not coded in STATA. Others, like the PCSE, FGLS, and seemingly unrelated regression (SUR), are feasible for T (the number of time series) > N (the number of cross-sectional units) [114, 115]. However, a DKSE estimate is feasible for N > T [114]. Therefore, depending on the CD, cointegration test, availability in STATA, and comparing N against T, this study mainly employs the DKSE regression for *models 1A and 1B*, FE model for *model 1C*, and GMM for *model 1C*.

Finally, to check the robustness of the main result, this study employs FMOLS, FGLS, and PCSE estimation techniques for *models 1A and 1B*. Furthermore, even though the Hausman test confirms that the FE is more efficient, the study employs the RE for *model 1C*. This is because Firebaugh et al. [116] note that the RE and FE models perform best in panel data. Besides, unlike FE, RE assumes that individual differences are random. In addition, this study uses panel DFE for *model 1D* (selected based on the Hausman test). Finally, the robustness check is also conducted using an alternative model (i.e., when a dependent variable is without a natural log and Granger causality test).

## Results

### Descriptive statistics and correlation analysis

Table 3 shows the overall mean of LNLEXP of the region is 4.063 years which indicates that the region can achieve only 57.43 (using ln(x) = 4.063 = loge^(x)^ = e^4.063^, where e = 2.718) years of life expectancy. This is very low compared to other regions. Besides, the ranges in the value of LNLEXP are between 3.698 and 4.345 or (40–76 years), implying high variation. Similarly, the mean value of LNINFMOR is 3.969; implying SSA countries recorded 52 infants death per 1000. Moreover, the range of LNINFMOR is between 2.525 and 4.919 or (12 – 135 infant death per 1000), implying high variation within the region. The mean value of people’s prevalence for undernourishment is 21.26; indicating 21% of the population is undernourished. However, the mean value of AVRDES is 107.826, which is greater than 100, implying that the calorie supply is adequate for all consumers if the food is distributed according to the requirements of individuals. When we observe the skewness and kurtosis of the variables of the models, except for LNLEXP and LNINFMOR, all variables are positively skewed. In addition, all variables have positive kurtosis with values between 2.202 and 6.092.

**Table 3:** Descriptive statistics and correlation analysis

Variables	Descriptive statistics						Correlation analysis							
	Mean	SD	Min	Max	Sk	Ku	1	2	3	4	5	6	7	8
1	4.063	0.125	3.698	4.345	-0.039	2.828	1	-0.742	-0.259	0.173	0.288	-0.028	0.317	-0.017
2	3.969	0.471	2.526	4.919	-0.925	3.995		1	0.390	-0.207	-0.449	-0.363	-0.600	-0.080
3	21.267	11.523	3.5	67.5	0.56	2.997			1	-0.899	-0.192	-0.045	-0.294	-0.324
4	107.826	11.796	74	135	0.081	2.203				1	0.155	-0.117	0.163	0.283
5	2418.088	2751.256	194.873	12064.78	1.634	4.546					1	0.264	0.642	0.374
6	1.82	1.225	-0.171	6.763	1.589	5.531						1	0.435	0.148
7	4.681	2.086	1.3	10.2	0.512	2.711							1	0.365
8	13.307	20.293	-0.151	88.559	2.012	6.092								1

Source: Computed by the author using STATA 15

*SD* Standard deviation, *Sk* Skewness, *Ku* Kurtosis, 1 = LNLEXP, 2 = LNINFMOR, 3 = PRUND, 4 = AVRDES, 5 = GDPPC, 6 = GOVEXP, 7 = MNSCHOOL, 8 = URBAN

Table 3 also shows the degree of relationship between variables, such that most values are below the threshold or rule of thumb (0.7) for a greater association [117]. However, the association between LNINFMOR and LNLEXP, as well as between PRUNP and AVRDES, is over the threshold and seems to have a multicollinearity issue. Nevertheless, these variables did not exist together in the models, indicating the absence of a multicollinearity problem.

### Cross-sectional and time-specific fixed effect

Table 4 shows whether the cross-sectional specific and time-specific FE in extended models (*model 1A-1D* plus Eq. 2) are valid. The result reveals that the null hypothesis of the captured unobserved heterogeneity is homogenous across the countries, and time is rejected at 1%, implying the extended models are correctly specified. Besides, to check the robustness of the two-way error component model relative to the pooled OLS estimator, this study conducted an additional poolability test. The result shows the null hypothesis that intercepts homogeneity (pooling) is rejected at a 1% level; thus, the FE model is most applicable, but the pooled OLS is biased.

**Table 4:** Test for individual cross-sections and time-specific effects

Tests	***Model 1A***		***Model 1B***		***Model 1C***		***Model 1D***		Decisions
	Test statistics	Prob	Test statistics	Prob	Test statistics	Prob	Test statistics	Prob	
Null hypothesis (H0): zero cross section and time effects: Poolability test (F-test)	F(47,505) = 134.41	0.0000	F(47,505) = 135.62	0.0000	F(47,505) = 148.62	0.0000	F(47,505) = 154.53	0.0000	reject H0 at 1% level
H0: Pooled OLS model is appropriate: Poolability test (F-test)	F(47,505) = 328.01	0.0000	F(47,505) = 266.86	0.0000	F(47,505) = 409.80	0.0000	F(47,505) = 446.03	0.0000	reject H0 at 1% level

Source: Computed by the author using STATA 15

### Detecting outliers and influential observations

Cooks D is an indicator of high leverage and residuals. The impact is high when D exceeds 4/N, (N = number of observations). A D > 1 implies a significant outlier problem. The Cooks D result of this study confirms the absence of outliers' problem (see supplementary file 1).

### Normality, heteroscedasticity, serial correlation, and multicollinearity tests

The results in Table 5 indicate that the probability value of the joint test for normality on e and u are above 0.01, implying that the residuals are normally distributed. The heteroscedasticity results show that the probability value of the chi-square statistic is less than 0.01 in all models. Therefore, the null hypothesis of constant variance can be rejected at a 1% level of significance. In other words, the modified Wald test result for Groupwise heteroskedasticity presented in Table 5, rejects the null hypothesis of Groupwise homoskedasticity observed by the probability value of 0.0000, which implies the presence of heteroscedasticity in the residuals. Similarly, all models suffer from serial correlation since the probability value of 0.0000 rejects the null hypothesis of no first-order serial correlation, indicating the presence of autocorrelation in all panel models. Finally, the multicollinearity test reveals that the models have no multicollinearity problem since the Variance inflation Factors (VIF) values are below 5.

**Table 5:** Normality, heteroskedasticity, serial correlation, and multicollinearity results

Tests		***Model 1A***	***Model 1B***	***Model 1C***	***Model 1D***
Normality	Joint test for Normality on e:	Chi2(2) = 1.66 Prob > chi2 = 0.4351	Chi2(2) = 1.28 Prob > chi2 = 0.5280	Chi2(2) = 1.42 Prob > chi2 = 0.4921	Chi2(2) = 1.44 Prob > chi2 = 0.4875
	Joint test for Normality on u:	Chi2(2) = 0.36 Prob > chi2 = 0.8370	Chi2(2) = 0.80 Prob > chi2 = 0.6699	Chi2(2) = 0.67 Prob > chi2 = 0.7160	Chi2(2) = 1.88 Prob > chi2 = 0.3905
Modified Wald test of GroupWise heteroskedasticity		Chi2 (31) = 22,514.01 Prob > chi2 = 0.0000	Chi2 (31) = 75,192.97 Prob > chi2 = 0.0000	Chi2 (31) = 20,940.21 Prob > chi2 = 0.0000	Chi2 (31) = 15,925.23 Prob > chi2 = 0.0000
Serial correlation		F(1,30) = 712.241 Prob > F = 0.0000	F(1,30) = 785.627 Prob > F = 0.0000	F(1,30) = 477.443 Prob > F = 0.0000	F(1,30) = 532.280 Prob > F = 0.0000
Multicollinearity/Variables		VIF			
MNSCHOOL		2.1	2.04	2.1	2.04
GDPPC		1.77	1.77	1.77	1.77
URBAN		1.28	1.27	1.28	1.27
GOVEXP		1.25	1.29	1.25	1.29
PRUND		1.18	-	1.18	-
AVRDES		-	1.14	-	1.14
Mean VIF		1.51	1.50	1.51	1.5

Source: Computed by the author using STATA 1

### Cross-sectional dependence test

Results in Table 6 strongly reject the null hypothesis of cross-sectional independence for *models 1A – 1C*. However, for *model 1D*, the study found mixed results (i.e., Pesaran [68] fails to reject the null hypothesis of no CD while Frees [72] strongly rejects it). Thus, to decide, this study employs the Friedman [71] CD test. The result fails to reject the null hypothesis of cross-sectional independence, implying that two out of three tests fail to reject the null hypothesis of cross-sectional independence in *model 1D*. Therefore, unlike others, there is no CD in *model 1D* (see Table 6).

**Table 6:** Cross-sectional dependence tests

	***Model 1A***		***Model 1B***		***Model 1C***		***Model 1D***	
tests	Statistics	Prob	Statistics	Prob	Statistics	Prob	Statistics	Prob
Pesaran’s test	8.862***	0.0000	5.971***	0.0000	3.673***	0.0000	0.148	0.8824
Frees’ test	10.067***	0.0000	10.309***	0.0000	8.010***	0.0000	8.386***	0.0000
Friedman’s test	-	-	-	-	-	-	16.254	0.9805

Source: Computed by the author using STATA 15

*CD* Cross-Sectional Dependence

^***^ *p* < 0.01

### Unit root tests

Table 7 shows that all variables are highly (at 1% level) significant either at level (I(0)) or first difference (I(1)), which implies all variables are stationary. In other words, the result fails to reject the null hypothesis of unit root (non-stationary) for all variables at a 1%-significance level, either at levels or the first differences. Thus, we might expect a long-run connection between these variables collectively.

**Table 7:** Unit root tests

Pesaran [68] unit root test (***Models 1A–1C***)								
Variables	CIPS (intercepts only)						Critical values (CV)	
	Levels			1st difference				
	Calculated Statistic	Prob	Calculated Statistic		Prob	10%	5%	1%
LNLEXP	-3.839***	< 1% (CV)	-2.854***		< 1% (CV)	-2.03	-2.11	-2.25
LNINFMOR	-1.884	> 10%	-2.548***		< 1% (CV)			
PRUDN	-1.528	> 10%	-2.280***		< 1% (CV)			
AVRDES	-2.110**	5%	-2.518***		< 1% (CV)			
GDPPC	-0.978	> 10%	-2.800***		< 1% (CV)			
GOVEXP	-1.550	> 10%	-3.925***		< 1% (CV)			
MNSCHOOL	-2.036*	10%	-4.070***		< 1% (CV)			
URBAN	-1.997	> 10%	-2.976***		< 1% (CV)			
***Model 1D***								
		Level			1^st^ difference			
LNINFMOR	Types	Statistic	Prob		Statistic	Prob	Order of integration	
	LLC	-5.7459***	0.0000		-	-	I(0)	
	IPS	-2.5397***	0.0055		-	-		
	ADF	463.0545***	0.0000		-	-		
AVRDES	LLC	-2.5844***	0.0049		-	-	I(0)	
	IPS	4.1243	1.0000		-5.7624***	0.0000	I(1)	
	ADF	63.9411	0.4082		121.1623***	0.0000	I(1)	
GDPPC	LLC	2.4703	0.9932		-13.1832***	0.0000		
	IPS	-1.7074**	0.0439		-12.3152***	0.0000	I(1)	
	ADF	59.1962	0.5775		301.7121***	0.0000		
GOVEXP	LLC	-1.2336	0.1087		-20.1958***	0.0000		
	IPS	0.0924	0.5368		-10.9719***	0.0000	I(1)	
	ADF	62.6224	0.4540		545.9615***	0.0000		
MNSCHOOL	LLC	-3.5101***	0.0002		-	-	I(0)	
	IPS	-0.6925	0.2443		-11.0645***	0.0000	I(1)	
	ADF	48.8314	0.8883		607.8821***	0.0000	I(1)	
URBAN	LLC	4.8714	1.0000		-19.7382***	0.0000		
	IPS	5.2350	1.0000		-10.5568***	0.0000	I(1)	
	ADF	74.0224	0.1410		378.0689***	0.0000		

Source: Computed by the author using STATA 15

*ADF* Augmented Dickey–Fuller, *AVRDES* Average Dietary Energy Supply, *CIPS* Cross-Sectionally Augmented Panel Unit Root Test, *GDPPC* Gross Domestic Product (GDP) per capita, *GOVEXP* Domestic General Government Health Expenditure, *I(1)* Integration at First Difference, *IPS* Im, Pesaran, Shin, *LLC* Levin, Lin, and Chu, *LNINFMOR* Natural Logarithm of Infant Mortality Rate, *LNLEXP* Natural Logarithm of Life Expectancy at Birth, *MNSCHOOL* Mean Years of Schooling, *PRUDN* Prevalence of Undernourishment, *URBAN* Urbanisation

^***^ *p* < 0.01

^**^ *p* < 0.05

^*^ *p* < 0.1

### Cointegration tests

The results in Table 8 show that both the Westerlund [98] and Banerjee and Carrion-i-Silvestre [104] cointegration tests strongly reject the null hypothesis of no-cointegration in *models 1A and 1B*. However, *model 1C* provides a mixed result, i.e. the Banerjee and Carrion-i-Silvestre [104] test rejects the null hypothesis of no cointegration, yet the reverse is true for the Westerlund [98] test. Therefore, this study conducted further cointegration tests for *model 1C*. Even though Westerlund and Edgerton [99] suffer from insufficient observation, it is based on the McCoskey and Kao [105] LM test [118]. Thus, we can use a residual-based cointegration test in the heterogeneous panel framework proposed by McCoskey and Kao [105]. However, an efficient estimation technique of cointegrated variables is required, and hence the FMOLS and DOLS estimators are recommended. The residuals derived from the FMOLS and DOLS will be tested for stationarity with the null hypothesis of no cointegration amongst the regressors. Since the McCoskey and Kao [105] test involves averaging the individual LM statistics across the cross-sections, for testing the residuals FMOLS and DOLS stationarity, McCoskey, and Kao [105] test is in the spirit of IPS (Im et al. [74]) [119].

**Table 8:** Panel cointegration test

Models	Values		Westerlund [98] tests for only target variables							Banerjee and Carrion-i-Silvestre [104] –for all variables in the model	Critical values		
			Gt		Ga		Pt	Pa		Levels Statistic	10%	5%	1%
***Model1A***	Z-value		-34.004*** (0.000)		-1.463* (0.072)		-74.878*** (0.000)	-34.439*** (0.000)		-4.062***	-2.03	-2.11	-2.25
	Bootstrap *P*-value		0.000***		0.000***		0.000***	0.000***					
***Model 1B***	Z-value		-34.708*** (0.000)		-4.303*** (0.000)		-52.636*** (0.000)	-37.899*** (0.000)		-4.212***			
	Bootstrap *P*-value		0.000***		0.000***		0.000***	0.000***					
***Model 1C***	Z-value		6.958 (1.000)		7.497 (1.000)		3.508 (1.000)	4.582 (1.000)		-2.907***			
	Bootstrap *P*-value		0.990		0.990		0.440	0.380					
Further cointegration tests **(** ***Model 1C)***													
DOLS residuals		Included variables		Unit root test		Statistic				*p*-value			
		between target variables		IPS		t-bar			-1.5321	0.9509	-1.82	-1.73	-1.69
						t-tilde-bar			-1.1501				
						Z-t-tilde-bar			1.6541				
		All variables in the model		IPS		t-bar			-1.2116	0.9984			
						t-tilde-bar			-0.9728				
						Z-t-tilde-bar			2.9494				
Pedroni [109] cointegrtaion test ***(Model 1D)***													
Test						Statistics				*p*-value			
Within-dimension			Modified variance ratio			-8.1614***				0.0000			
			Modified Phillips-Perron t			2.7529***				0.0030			
			Phillips-Perron t			-6.0649***				0.0000			
			Augmented Dickey-Fuller t			-5.0621***				0.0000			
Between-dimension			Modified Phillips-Perron t			5.9229***				0.0000			
			Phillips-Perron t			-2.2026**				0.0138			
			Augmented Dickey-Fuller t			-2.0690**				0.0193			

Source: Computed by the author using STATA 15

*P*-values in parentheses

^***^ *p* < 0.01

^**^ *p* < 0.05

^*^ *p* < 0.1

Though FMOLS and DOLS are recommended for the residuals cointegration test, DOLS is better than FMOLS (for more detail, see Kao and Chiang [120]); therefore, this study uses a residual test derived from DOLS. The result fails to reject the null hypothesis of no cointegration. Two (Banerjee and Carrion-i-Silvestre [104] and McCoskey and Kao [105]) out of three tests fail to reject the null hypothesis of no cointegration; hence, we can conclude that there is no long-run relationship among the variables in *model 1C*.

Unlike other models, since there is CD in *model 1D*, this study employs the Pedroni [109] and Kao [111] cointegration tests for *model 1D*. The result strongly rejects the null hypothesis of no cointegration, which is similar to *models 1A and 1B*, that a long-run relationship exists among the variables in *model 1D* (see Table 5).

## Panel data estimation results

Table 9 provides long-run regression results of all models employing appropriate estimation techniques such as DKSE, FE, and two-step GMM, along with the Granger causality test. However, the DKSE regression can be estimated in three ways: FE with DKSE, RE with DKSE, and pooled Ordinary Least Squares/Weighted Least Squares (pooled OLS/WLS) regression with DKSE. Hence, we must choose the most efficient model using Hausman and Breusch-Pagan LM for RE tests (see supplementary file 2). As a result, this study employed FE with DKSE for *models 1A and 1B*. Further, due to Hausman's result, absence of cointegration and to deal with heterogeneity and spatial dependence in the dynamic panel, this study employs FE for the *model1C* (see the supplementary file 2). However, due to the absence of CD, the presence of cointegration, and N > T, this study uses GMM for *model 1D*. Moreover, according to Roodman [121], the GMM approach can solve heteroskedasticity and autocorrelation problems. Furthermore, even though two-step GMM produces only short-run results, it is possible to generate long-run coefficients from short-run results [122, 123].

**Table 9:** DKSE, FE, Two-step GMM, and Granger causality results

	Coef	Std. Err	t	P > t	[95% Conf. Interval]		Other statistics	
**Model 1A using DKSE when the dependent variable is LNLEXP**								
PRUND	-0.00348***	0.0008573	-4.06	0.001	-0.005289	-0.0016715	F(5,17) = 435.58 Prob > F = 0.0000 within R^2^ = 0.6434	
GDPPC	-4.28e-06	2.95e-06	-1.45	0.165	-0.0000105	1.95e-06		
GOVEXP	0.00474	0.0049357	0.96	0.350	-0.0056726	0.0151541		
MNSCHOOL	0.0936***	0.004993	18.76	0.000	0.0831156	0.1041842		
URBAN	-0.00011	0.0001784	-0.65	0.526	-0.0004919	0.0002609		
_CONS	3.7019***	0.0371405	99.67	0.000	3.623589	3.780308		
**Model 1B using DKSE when the dependent variable is LNLEXP**								
AVRDES	0.00317***	0.000845	3.76	0.002	0.0013936	0.0049593	F(5,17) = 410.16 Prob > F = 0.0000 within R^2^ = 0.6295	
GDPPC	-3.81e-06	2.78e-06	-1.37	0.188	-0.0000097	0.0000021		
GOVEXP	0.00188	0.00573	0.33	0.746	-0.0102006	0.0139790		
MNSCHOOL	0.0926***	0.005319	17.42	0.000	0.0814219	0.1038642		
URBAN	-0.00011	0.000166	-0.67	0.512	-0.0004620	0.0002392		
_CONS	3.2941***	0.074949	43.95	0.000	3.1360170	3.4522750		
**Model 1C using the FE model when the dependent variable is LNINFMOR**								
PRUND	0.0119***	0.0009722	12.29	0.000	0.010040	0.013860	sigma_u = 0.45608378 sigma_e = 0.10751418 F test that all u_i = 0: F(30, 522) = 164.49 Prob > F = 0.0000 within R^2^ = 0.6823	
GDPPC	0.000057***	0.0000087	6.53	0.000	0.000040	0.000074		
GOVEXP	-0.0103	0.0088257	-1.17	0.241	-0.027691	0.006986		
MNSCHOOL	-0.2812***	0.0110858	-25.37	0.000	-0.303052	-0.259495		
URBAN	0.00061	0.0006116	1.00	0.316	-0.000588	0.001815		
_CONS	4.9049***	0.0575012	85.30	0.000	4.791987	5.017912		
**Model 1D using GMM when the dependent variable is LNINFMOR**								
L_LNINFMOR	32.1844***	10.5261	3.06	0.002	11.55363	52.81518		No of instruments = 21, No of groups = 31 AR(1): z = -0.98 Pr > z = 0.326 AR(2): z = -1.02 Pr > z = 0.308 Sargan test of over-identification: Chi2(15) = 4.15 Prob > chi2 = 0.997 Hansen test of over-identification: Chi2(15) = 12.22 Prob > chi2 = 0.662
AVRDES	-0.0139***	0.0052795	-2.63	0.008	-0.024255	-0.00356		
GDPPC	0.000065	0.000082	0.8	0.424	-0.0000952	0.000226		
GOVEXP	-0.0891**	0.0372889	-2.39	0.017	-0.1622396	-0.01607		
MNSCHOOL	-0.1152	0.1239816	-0.93	0.352	-0.3582814	0.127717		
URBAN	0.0010	0.0013938	0.75	0.454	-0.0016875	0.003776		
**Dumitrescu and Hurlin** [124]** Granger causality test**								
Null hypothesis			W-bar	Z-bar	Z-bar tilde	p-value		Decision
PRUND does not Granger-cause LNLEXP			17.5322	65.0874	48.4371	0.0000		PRUND does Granger-cause LNLEXP
AVRDES does not Granger-cause LNLEXP			13.2728	48.3179	35.8304	0.0000		AVRDES does Granger-cause LNLEXP
PRUND does not Granger-cause LNINFMOR			5.0078	15.7787	11.3686	0.0000		PRUND does Granger-cause LNINFMOR
AVRDES does not Granger-cause LNINFMOR			5.2776	16.8409	12.1671	0.0000		AVRDES does Granger-cause LNINFMOR

Source: Computed by the author using STATA 15

*AVRDES* Average Dietary Energy Supply, *DKSE* Driscoll-Kraay Standard Errors, *FE* Fixed Effect, *GDPPC* Gross Domestic Product (GDP) per capita, *GMM* Generalised Method of Momentum, *GOVEXP* Domestic General Government Health Expenditure, *L_LNINFMOR* Lag of Natural Logarithm of Infant Mortality Rate, *LNINFMOR* Natural Logarithm of Infant Mortality Rate, *LNLEXP* Natural Logarithm of Life Expectancy at Birth, *MNSCHOOL* Mean Years of Schooling, *PRUDN* Prevalence of Undernourishment, *URBAN* Urbanisation

^***^ *p* < 0.01

^**^ *p* < 0.05

The DKSE result of *model 1A* shows that a 1% increment in people's prevalence for undernourishment reduces their life expectancy by 0.00348 PPs (1 year or 366 days). However, in *model 1C,* a 1% rise in the prevalence of undernourishment increases infant mortality by 0.0119 PPs (1 year or 369 days). The DKSE estimations in model 1B reveal that people’s life expectancy rises by 0.00317 PPs with every 1% increase in average dietary energy supply. However, the GMM result for *model 1D* confirms that a 1% incrementin average dietary energy supply reduces infant mortality by 0.0139 PPs. Moreover, this study conducted a panel Granger causality test to confirm whether or not food insecurity has a potential causality to health outcomes. The result demonstrates that the null hypothesis of change in people’s prevalence for undernourishment and average dietary energy supply does not homogeneously cause health outcomes is rejected at 1% significance, implying a change in food insecurity does Granger-cause health outcomes of SSA countries (see Table 9).

In addition to the main results, Table 9 also reports some post-estimation statistics to ascertain the consistency of the estimated results. Hence, in the case of DKSE and FE models, the validity of the models is determined by the values of R^2^ and the F statistics. For instance, R^2^ quantifies the proportion of the variance in the dependent variable explained by the independent variables, representing the model’s quality. The results in Table 9 demonstrate that the explanatory variables explain more than 62% of the variance on the dependent variable. Cohen [125] classifies the R^2^ value of 2% as a moderate influence in social and behavioral sciences, while 13 and 26% are considered medium and large effects, respectively. Therefore, the explanatory variables substantially impact this study's models. Similarly, the F statistics explain all independent variables jointly explain the dependent one. For the two-step system GMM, the result fails to reject the null hypothesis of no first (AR(1)) and second-order (AR(2)) serial correlation, indicating that there is no first and second-order serial correlation. In addition, the Hansen [126] and Sargan [127] tests fail to reject the null hypothesis of the overall validity of the instruments used, which implies too many instruments do not weaken the model.

### Robustness checks

The author believes the above findings may not be enough for policy recommendations unless robustness checks are undertaken. Hence, the study estimated all models without the natural logarithm of the dependent variables (see Table 10). The *model 1A* result reveals, similar to the above results, individuals’ prevalence for undernourishment significantly reduces their life expectancy in SSA countries. That means a 1% increase in the people's prevalence of undernourishment reduces their life expectancy by 0.1924 PPs. Moreover, in *model 1B*, life expectancy rises by 0.1763 PPs with every 1% increase in average dietary energy supply. In *model 1C*, the rise in infants’ prevalence for undernourishment has a positive and significant effect on their mortality rate in SSA countries. The FE result implies that a 1% rise in infants’ prevalence for undernourishment increases their mortality rate by 0.9785 PPs. The GMM result in *model 1D* indicates that improvement in average dietary energy supply significantly reduces infant mortality. Further, the Granger causality result confirms that the null hypothesis of change in the prevalence of undernourishment and average dietary energy supply does not homogeneously cause health outcomes and is rejected at a 1% level of significance. This implies a change in food insecurity does Granger-cause health outcomes in SSA countries (see Table 10).

**Table 10:** DKSE, FE, Two-step GMM, and Granger causality results

	Coef	Std. Err	t	P > t	[95% Conf. Interval]		Other statistics
**Model 1A using DKSE when the dependent variable is LEXP**							
PRUND	-0.1924***	0.042083	-4.57	0.000	-0.28121	-0.10363	F(5,17) = 660.10 Prob > F = 0.0000 within R^2^ = 0.6603
GDPPC	-0.00023	0.000157	-1.43	0.170	-0.00056	0.000107	
GOVEXP	0.2805	0.264204	1.06	0.303	-0.27686	0.837985	
MNSCHOOL	5.3427***	0.305739	17.47	0.000	4.697669	5.987775	
URBAN	-0.0107	0.010292	-1.04	0.311	-0.03245	0.010973	
_ CONS	37.8656***	2.066197	18.33	0.000	33.50632	42.22491	
**Model 1B using DKSE when the dependent variable is LEXP**							
AVRDES	0.1762***	0.041312	4.27	0.001	0.089105	0.263427	F(5,17) = 594.21 Prob > F = 0.0000 within R^2^ = 0.6472
GDPPC	-0.0002	0.000148	-1.35	0.194	-0.00051	0.000112	
GOVEXP	0.1238	0.304085	0.41	0.689	-0.51776	0.765368	
MNSCHOOL	5.2847***	0.322442	16.39	0.000	4.604474	5.965058	
URBAN	-0.0105	0.009715	-1.08	0.293	-0.03103	0.009964	
_CONS	15.2589***	3.5383	4.31	0.000	7.793746	22.72407	
**Model 1C using FE when the dependent variable is INFMOR**							
PRUND	0.9785***	0.057732	16.95	0.000	0.86511	1.091941	sigma_u = 25.168803 sigma_e = 6.3846649 F test that all u_i = 0: F(30, 522) = 126.57 Prob > F = 0.0000 within R^2^ = 0.6888
GDPPC	0.0035***	0.000517	6.88	0.000	0.00254	0.004569	
GOVEXP	-0.3123	0.52411	-0.6	0.551	-1.34201	0.717236	
MNSCHOOL	-15.1801***	0.658325	-23.06	0.000	-16.4734	-13.8868	
URBAN	-0.0132	0.036319	-0.37	0.715	-0.08462	0.058075	
_CONS	100.7214***	3.414677	29.5	0.000	94.01316	107.4296	
**Model 1D using GMM when the dependent variable is INFMOR**							
L_INFMOR	22.6035***	5.349301	4.23	0.000	12.11909	33.08797	No of instruments = 21, No of groups = 31 AR(1): z = -1.37 Pr > z = 0.170 AR(2): z = 0.30 Pr > z = 0.768 Sargan test of over-identification: Chi2(15) = 6.80 Prob > chi2 = 0.963 Hansen test of over-identification: Chi2(15) = 17.36 Prob > chi2 = 0.298
AVRDES	-0.2809	0.175223	-1.6	0.109	-0.6244	0.062461	
GDPPC	-0.0038**	0.001689	-2.27	0.023	-0.00715	-0.00053	
GOVEXP	-3.2419***	1.057435	-3.07	0.002	-5.31445	-1.16938	
MNSCHOOL	6.8813	6.48081	1.06	0.288	-5.82083	19.58348	
URBAN	0.0955*	0.057111	1.67	0.094	-0.01641	0.20746	
**Dumitrescu and Hurlin** [124]** Granger causality test**							
Null hypothesis		W-bar		Z-bar	Z-bar tilde	p-value	Decision
PRUND does not Granger-cause LEXP		18.3305		68.2301	50.7996	0.0000	PRUND does Granger-cause LEXP
AVRDES does not Granger-cause LEXP		14.2891		52.3192	38.8384	0.0000	AVRDES does Granger-cause LEXP
PRUND does not Granger-cause INFMOR		5.4921		17.6854	12.8020	0.0000	PRUND does Granger-cause INFMOR
AVRDES does not Granger-cause INFMOR		5.7553		18.7214	13.5808	0.0000	AVRDES does Granger-cause INFMOR

Source: Computed by the author using STATA 15

*AVRDES* Average Dietary Energy Supply, *DKSE* Driscoll-Kraay Standard Errors, *FE* Fixed Effect, *GMM* Generalised Method of Momentum, *INFMOR* Infant Mortality Rate, *L_INFMOR* Lag of Infant Mortality Rate, *LEXP* Life Expectancy at Birth, *PRUDN* Prevalence of Undernourishment

^***^ *p* < 0.01

^**^ *p* < 0.05

^*^ *p* < 0.1

The study also conducted further robustness checks using the same dependent variables (as Table 9) but different estimation techniques. The results confirm that people’s prevalence of undernourishment has a negative and significant effect on their life expectancy, but improvement in average dietary energy supply significantly increases life expectancy in SSA countries. However, the incidence of undernourishment in infants contributes to their mortality; however, progress in average dietary energy supply for infants significantly reduces their mortality (see Table 11).

**Table 11:** FMOLS, PCSE, FGLS, RE, and DFE results

Variables	FMOLS		PCSE		FGLS		RE	DFE
	Dependent variable (LNLEXP)						Dependent variable (LNINFMOR)	
	***Model 1A***	***Model 1B***	***Model 1A***	***Model 1B***	***Model 1A***	***Model 1B***	***Model 1C***	***Model 1D***
PRUDN	0.0452*** (0.0005647)	-	-0.0024*** (0.0002788)	-	-0.0023*** (0.0000129)	-	0.0120*** (0.0009780)	-
AVRDES	-	0.0510*** (0.0013406)	-	0.0013*** (0.0003409)	-	0.0013*** (0.0000168)	-	-0.0177*** (0.0055162)
GDPPC	-0.00036*** (0.0000433)	0.00044*** (0.0000956)	0.0000089*** (0.0000008)	0.0000082*** (0.0000008)	0.0000089*** (0.0000001)	0.0000083*** (0.0000001)	0.000053*** (0.0000085)	0.000092* (0.0000491)
GOVEXP	0.2341*** (0.0068580)	-0.2476*** (0.0081426)	-0.0184*** (0.0033015)	-0.0176*** (0.0031635)	-0.0180*** (0.0002090)	-0.0172*** (0.0002151)	-0.0117 (0.0089028)	-0.1371** (0.0592705)
MNSCHOOL	0.6685*** (0.0076138)	-0.3046*** (0.0372598)	0.0175*** (0.0016869)	0.0200*** (0.0017382)	0.0175*** (0.0001854)	0.0200*** (0.0001456)	-0.2669*** (0.0108812)	-0.0427 (0.0821548)
URBAN	-0.00139*** (0.0001734)	0.0026*** (0.0003066)	-0.0014*** (0.0001823)	-0.0013*** (0.0001775)	-0.0014*** (0.0000421)	-0.0013*** (0.0000404)	0.0010* (0.0006106)	0.0020 (0.0040979)
_CONS	-	-	4.0639*** (0.0071382)	3.8493*** (0.0462690)	4.0625*** (0.0009768)	3.8502*** (0.0021131)	4.8416*** (0.0838447)	0.1843*** (0.0544606)
ECM	-	-	-	-	-	-	-	-0.0352*** (0.0080052)
	R^2^ = 0.71880.7188944	R^2^ = 0.4593	Wald chi2(5) = 1072.48 Prob > chi2 = 0.0000 R^2^ = 0.2165	Wald chi2(5) = 1019.33 Prob > chi2 = 0.0000 R^2^ = 0.1896	Waldchi2(5) = 52,866.78Prob > chi2 = 0.0000	Wald chi2(5) = 124,327.41Prob > chi2 = 0.0000	sigma_u = 0.3413 sigma_e = 0.1075 Wald chi2(5) = 1078.43 Prob > chi2 = 0.0000	

Source: Computed by the author using STATA 15

*AVRDES* Average Dietary Energy Supply, *DFE* Dynamic Fixed Effect, *ECM* Error Correction Model, *FGLS* Feasible Generalised Least Squares, *FMOLS* Fully Modified Ordinary Least Square, *LNINFMOR* Natural Logarithm of Infant Mortality Rate, *LNLEXP* Natural Logarithm of Life Expectancy at Birth, *PCSE* Panel-Corrected Standard Error, *PRUDN* Prevalence of Undernourishment, *RE* Random Effect

Standard errors in parentheses

^***^ *p* < 0.01

^**^ *p* < 0.05

^*^ *p* < 0.1

## Discussion

The main objective of this study is to examine the impact of food insecurity on the health outcomes of SSA countries. Accordingly, the DKSE result of *model 1A* confirms that the rise in people’s prevalence for undernourishment significantly reduces their life expectancy in SSA countries. However, the FE result shows that an increment in the prevalence of undernourishment has a positive and significant impact on infant mortality in *model 1C*. This indicates that the percentage of the population whose food intake is insufficient to meet dietary energy requirements is high, which leads to reduce life expectancy but increases infant mortality in SSA countries. The reason for this result is linked to the insufficient food supply in SSA due to low production and yields, primitive tools, lack of supporting smallholder farms and investment in infrastructure, and government policies. Besides, even though the food is available, it is not distributed fairly according to the requirements of individuals. Moreover, inadequate access to food, poor nutrition, and chronic illnesses are caused by a lack of well-balanced diets. In addition, many of these countries are impacted by poverty, making it difficult for citizens to afford nutritious food. All these issues combine to create an environment where individuals are more likely to suffer malnutrition-related illnesses, resulting in a lower life expectancy rate. The DKSE estimation result in *model 1B* reveals that improvement in average dietary energy supply positively impacts people's life expectancy in SSA countries. However, the improvement in average dietary energy supply reduces infant mortality.

Based on the above results, we can conclude that food insecurity harms SSA nations' health outcomes. This is because the prevalence of undernourishment leads to increased infant mortality by reducing the vulnerability, severity, and duration of infectious diseases such as diarrhea, pneumonia, malaria, and measles. Similarly, the prevalence of undernourishment can reduce life expectancy by increasing the vulnerability, severity, and duration of infectious diseases. However, food security improves health outcomes – the rise in average dietary energy supply reduces infant mortality and increases the life expectancy of individuals.

Several facts and theories support the above findings. For instance, similar to the theoretical and conceptual framework section, food insecurity in SSA countries can affect health outcomes in nutritional, mental health, and behavioral channels. According to FAO et al. [128], the prevalence of undernourishment increased in Africa from 17.6% of the population in 2014 to 19.1% in 2019. This figure is more than twice the global average and the highest of all regions of the world. Similarly, SSA is the world region most at risk of food insecurity [129]. According to Global Nutrition [130] report, anemia affects an estimated 39.325% of women of reproductive age. Some 13.825% of infants have a low weight at birth in the SSA region. Excluding middle African countries (due to lack of data), the estimated average prevalence of infants aged 0 to 5 months who are exclusively breastfed is 35.73%, which is lower than the global average of 44.0%. Moreover, SSA Africa still experiences a malnutrition burden among children aged under five years. The average prevalence of overweight is 8.15%, which is higher than the global average of 5.7%. The prevalence of stunting is 30.825%—higher than the worldwide average of 22%. Conversely, the SSA countries’ prevalence of wasting is 5.375%, which is higher than most regions such as Central Asia, Eastern Asia, Western Asia, Latin America and the Caribbean, and North America. The SSA region's adult population also faces a malnutrition burden: an average of 9.375% of adult (aged 18 and over) women live with diabetes, compared to 8.25% of men. Meanwhile, 20.675% of women and 7.85% of men live with obesity.

According to Saltzman et al. [17], micronutrient deficiencies can affect people’s health throughout their life cycle. For instance, at the baby age, it causes (low birth weight, higher mortality rate, and impaired mental development), child (stunting, reduced mental capacity, frequent infections, reduced learning capacity, higher mortality rate), adolescent (stunting, reduced mental capacity, fatigue, and increased vulnerability to infection), pregnant women (increased mortality and perinatal complications), adult (reduced productivity, poor socio-economic status, malnutrition, and increased risk of chronic disease), elderly (increased morbidity (including osteoporosis and mental impairment), and higher mortality rate).

Though this study attempts to fill the existing gaps, it also has limitations. It examined the impact of food insecurity on infant mortality; however, their association is reflected indirectly through other health outcomes. Hence, future studies can extend this study by examining the indirect effect of food insecurity on infant mortality, which helps to look at in-depth relationships between the variables. Moreover, this study employed infant mortality whose age is below one year; hence, future studies can broaden the scope by decomposing infant mortality into (neonatal and postnatal) and under-five mortality.

## Conclusions

Millions of people are dying every year due to hunger and hunger-related diseases worldwide, especially in SSA countries. Currently, the link between food insecurity and health status is on researchers' and policymakers' agendas. However, macro-level findings in this area for most concerned countries like SSA have been given only limited attention. Therefore, this study examined the impact of food insecurity on life expectancy and infant mortality rates. The study mainly employs DKSE, FE, two-step GMM, and Granger causality approaches, along with other estimation techniques for robustness checks for the years between 2001 and 2018. The result confirms that food insecurity harms health outcomes, while food security improves the health status of SSA nations'. That means that a rise in undernourishment increases the infant mortality rate and reduces life expectancy. However, an improvement in the average dietary energy supply reduces infant mortality and increases life expectancy. Therefore, SSA countries need to guarantee their food accessibility both in quality and quantity, which improves health status. Both development experts and political leaders agree that Africa has the potential for agricultural outputs, can feed the continent, and improve socio-economic growth. Besides, more than half of the world's unused arable land is found in Africa. Therefore, effective utilization of natural resources is essential to achieve food security. Moreover, since the majority of the food in SSA is produced by smallholder farmers [131] while they are the most vulnerable to food insecurity and poverty [132, 133]; hence, special focus and support should be given to smallholder farmers that enhance food self-sufficiency. Further, improvement in investment in agricultural research; improvement in markets, infrastructures, and institutions; good macroeconomic policies and political stability; and developing sub-regional strategies based on their agroecological zone are crucial to overcoming food insecurity and improving health status. Finally, filling a stomach is not sufficient; hence, a person's diet needs to be comprehensive and secure, balanced (including all necessary nutrients), and available and accessible. Therefore, SSA countries should ensure availability, accessibility, usability, and sustainability to achieve food and nutrition security.

## Supplementary Information

**Additional file 1:** **Table S1.** Cook’s D results

**Additional file 2:** **Table S2.** Hausman and Breusch-Pagan LM for REtests. 

**Additional file 3.** 

## Data Availability

The datasets used and/or analyzed during the current study are available in supplementary materials.

## Abbreviations

ADF: Augmented Dickey–Fuller
AIDS: Acquired Immunodeficiency Syndrome
AVRDES: Average Dietary Energy Supply
CCEMG: Common Correlated Effects Mean Group
CCEP: Common Correlated Effects Pooled
CD: Cross-Sectional Dependence
CIPS: Cross-Sectionally Augmented Panel Unit Root Test
CS-ARDL: Cross-Section Augmented Autoregressive Distributed Lag
CS-DL: Cross-Section Augmented Distributed Lag
CUP-BC: Continuously Updated Bias-Corrected
CUP-FM: Continuously Updated Full Modified
DFE: Dynamic Fixed Effect
DKSE: Driscoll-Kraay Standard Errors
DOLS: Dynamic Ordinary Least Square
ECM: Error Correction Model
FAO: Food and Agricultural Organization
FE: Fixed Effect
FGLS: Feasible Generalised Least Squares
FMOLS: Fully Modified Ordinary Least Square
GDPPC: Gross Domestic Product (GDP) per capita
GMM: Generalised Method of Momentum
GOVEXP: Domestic General Government Health Expenditure
HIV: Human Immunodeficiency Virus
I(1): Integration at First Difference
IFAD: International Fund for Agricultural Development
INFMOR: Infant Mortality Rate
IPS: Im, Pesaran, Shin
L_INFMOR: Lag of Infant Mortality Rate
L_LNINFMOR: Lag of Natural Logarithm of Infant Mortality Rate
LEXP: Life Expectancy at Birth
LLC: Levin, Lin, and Chu
LM: Lagrange Multiplier
LNINFMOR: Natural Logarithm of Infant Mortality Rate
LNLEXP: Natural Logarithm of Life Expectancy at Birth
MG: Mean Group
MNSCHOOL: Mean Years of Schooling
OLS: Ordinary Least Squares
PCSE: Panel-Corrected Standard Error
PMG: Pooled Mean Group
PRUND: Prevalence of Undernourishment
RE: Random Effect
SDGs: Sustainable Development Goals
SSA: Sub-Saharan African
STATA: Statistical Software
SUR: Seemingly Unrelated Regression
URBAN: Urbanisation
WFP: World Food Programme
WHO: World Health Organization
WLS: Weighted Least Squares
