# Supplementary material for: The impact of food insecurity on health outcomes: empirical evidence from sub-Saharan African countries
Source: BMC Public Health. 2023 Feb 15;23:338. doi: 10.1186/s12889-023-15244-3 (PMC9930357; doi:10.1186/s12889-023-15244-3)
Supplement: Supplementary file 1 — Additional file 1: Table S1. Cook’s D results [file 12889_2023_15244_MOESM1_ESM.docx]

**Supplementary files**

**Supplementary file 1**

Table S1: Cook’s D results

| ***Model 1A*** | | ***Model 1B*** | | ***Model 1C*** | | ***Model 1D*** | |
| --- | --- | --- | --- | --- | --- | --- | --- |
| country | D | country | D | country | D | country | D |
| 8 | .0081805 | 8 | .0075835 | 3 | .008727 | 1 | .0082589 |
| 8 | .0076295 | 8 | .0074915 | 6 | .0082366 | 3 | .0090701 |
| 8 | .0081606 | 8 | .0077352 | 15 | .0112259 | 6 | .0080019 |
| 8 | .007339 | 15 | .0089001 | 15 | .0103089 | 15 | .0083997 |
| 8 | .0078778 | 15 | .0150657 | 15 | .0144807 | 15 | .0075404 |
| 15 | .0087792 | 15 | .0123562 | 15 | .011639 | 15 | .0114668 |
| 15 | .0081881 | 15 | .0146526 | 15 | .0167295 | 15 | .0087365 |
| 15 | .0111146 | 15 | .0110427 | 15 | .0189666 | 15 | .0130229 |
| 15 | .0173009 | 15 | .0113699 | 15 | .0176571 | 15 | .0152118 |
| 15 | .0145038 | 15 | .0093959 | 15 | .0182461 | 15 | .0142235 |
| 15 | .0163728 | 16 | .0085741 | 15 | .0089944 | 15 | .016593 |
| 15 | .0128153 | 16 | .0078739 | 15 | .0082457 | 15 | .0109485 |
| 15 | .0130645 | 16 | .0092533 | 15 | .0098539 | 15 | .0099905 |
| 15 | .0107362 | 16 | .0074907 | 15 | .0132624 | 15 | .0121748 |
| 15 | .0071987 | 16 | .0083744 | 16 | .019786 | 15 | .014684 |
| 16 | .0075264 | 26 | .0072985 | 16 | .0146495 | 16 | .0213008 |
| 16 | .0078619 | 26 | .0073323 | 16 | .0163776 | 16 | .0166584 |
| 24 | .0075226 | 26 | .0080372 | 16 | .0145905 | 16 | .018719 |
| 24 | .0079385 | 27 | .011124 | 16 | .0156565 | 16 | .0166383 |
| 26 | .0075237 | 27 | .0091313 | 16 | .0148321 | 16 | .0179412 |
| 27 | .0122834 |  |  | 16 | .0151413 | 16 | .0172144 |
| 27 | .0098204 |  |  | 16 | .016196 | 16 | .0175417 |
| 27 | .0074793 |  |  | 16 | .012063 | 16 | .0185128 |
|  |  |  |  | 16 | .0123624 | 16 | .0137611 |
|  |  |  |  | 21 | .0087308 | 16 | .0139862 |
|  |  |  |  | 21 | .0096058 | 21 | .009429 |
|  |  |  |  | 21 | .0106991 | 21 | .0103112 |
|  |  |  |  | 21 | .0107507 | 21 | .0116572 |
|  |  |  |  | 21 | .0109744 | 21 | .0120052 |
|  |  |  |  | 21 | .0115689 | 21 | .0121884 |
|  |  |  |  | 21 | .0125823 | 21 | .0127986 |
|  |  |  |  | 21 | .0133847 | 21 | .0138081 |
|  |  |  |  | 21 | .0131427 | 21 | .0147742 |
|  |  |  |  | 21 | .0113205 | 21 | .0145731 |
|  |  |  |  | 21 | .0112008 | 21 | .0128815 |
|  |  |  |  | 21 | .0098869 | 21 | .0124798 |
|  |  |  |  | 21 | .0088453 | 21 | .011095 |
|  |  |  |  | 21 | .0074555 | 21 | .0099844 |
|  |  |  |  | 21 | .008386 | 21 | .0085958 |
|  |  |  |  | 21 | .0071726 | 21 | .0095782 |
|  |  |  |  | 26 | .0072331 | 21 | .0083372 |
|  |  |  |  | 26 | .0076621 | 21 | .0075084 |
|  |  |  |  | 26 | .0082538 | 26 | .0076253 |
|  |  |  |  | 28 | .0081795 | 26 | .0076077 |
|  |  |  |  | 28 | .0075313 | 26 | .0077314 |
|  |  |  |  |  |  | 26 | .0084652 |
|  |  |  |  |  |  | 26 | .0088524 |
|  |  |  |  |  |  | 26 | .0093817 |

Note: 1= Angola, 3= Botswana, 6=Cabo Verde, 8= Congo, Rep., 15= Lesotho, 16= Liberia, 21=Mauritius, 24= Nigeria, 26=Senegal, 27=Sierra Leone, 28=South Africa.

Source: Computed by the author using STATA 15
