# Supplementary material for: The impact of food insecurity on health outcomes: empirical evidence from sub-Saharan African countries
Source: BMC Public Health. 2023 Feb 15;23:338. doi: 10.1186/s12889-023-15244-3 (PMC9930357; doi:10.1186/s12889-023-15244-3)
Supplement: Supplementary file 2 — Additional file 2: Table S2. Hausman and Breusch-Pagan LM for REtests. [file 12889_2023_15244_MOESM2_ESM.docx]

**Supplementary file 2**

Table S2: Hausman and Breusch-Pagan LM for RE tests

| Tests | ***Model 1A*** | ***Model 1B*** | ***Model 1C*** | ***Model 1D*** | Decision rule |
| --- | --- | --- | --- | --- | --- |
| Hausman | Chi2(5)=63.36  Prob>chi2=0.0000 | Chi2(5)= 55.89  Prob>chi2 = 0.0000 | Chi2(5)= 25.17  Prob>chi2=0.0001 | – | If the prob. value <0.05, FE is efficient than RE model |
| Breusch and Pagan LM | Chibar2(01)=2580.20  Prob>chibar2=0.0000 | Chibar2(01)=2629.62  Prob>chibar2=0.0000 | Chibar2(01)= 3286.59  Prob>chibar2= 0.0000 | – | If the prob. value<0.05, RE is efficient than pooled OLS. |
| Hausman | – | – | – | Chi2(5) = 0.87  Prob>chi2= 0.9721 | If the prob. value>0.05, pmg is efficient than mg model |
| Hausman | – | – | – | Chi2(5) = 0.00  Prob>chi2 = 1.0000 | If the prob. value>0.05, dfe is efficient than mg model |
| Hausman | – | – | – | Chi2(5) = 0.02  Prob>chi2 = 1.0000 | If the prob. value>0.05, dfe is efficient than pmg model |

Source: Computed by the author using STATA 15
